# A Dual Conductance Sensor for Simultaneous Measurement of Void Fraction and Structure Velocity of Downward Two-Phase Flow in a Slightly Inclined Pipe

**DOI:** 10.3390/s17051063

**Published:** 2017-05-08

**Authors:** Yeon-Gun Lee, Woo-Youn Won, Bo-An Lee, Sin Kim

**Affiliations:** 1 Department of Nuclear and Energy Engineering, Jeju National University, Jeju 63243, Korea; yeongun2@jejunu.ac.kr (Y.-G.L.); yuniwon@jejunu.ac.kr (W.-Y.W.); 2 Institute for Nuclear Science and Technology, Jeju National University, Jeju 63243, Korea; boani@jejunu.ac.kr; 3 School of Energy Systems Engineering, Chung-Ang University, Seoul 06974, Korea

**Keywords:** dual conductance sensor, inclined pipe, void fraction, structure velocity, flow regime

## Abstract

In this study, a new and improved electrical conductance sensor is proposed for application not only to a horizontal pipe, but also an inclined one. The conductance sensor was designed to have a dual layer, each consisting of a three-electrode set to obtain two instantaneous conductance signals in turns, so that the area-averaged void fraction and structure velocity could be measured simultaneously. The optimum configuration of the electrodes was determined through numerical analysis, and the calibration curves for stratified and annular flow were obtained through a series of static experiments. The fabricated conductance sensor was applied to a 45 mm inner diameter U-shaped downward inclined pipe with an inclination angle of 3° under adiabatic air-water flow conditions. In the tests, the superficial velocities ranged from 0.1 to 3.0 m/s for water and from 0.1 to 18 m/s for air. The obtained mean void fraction and the structure velocity from the conductance sensor were validated against the measurement by the wire-mesh sensor and the cross-correlation technique for the visualized images, respectively. The results of the flow regime classification and the corresponding time series of the void fraction at a variety of flow velocities were also discussed.

## 1. Introduction

Measurement techniques for gas-liquid two-phase flows are a challenging issue in a variety of industrial applications due to their irregular flow behavior as a result of the flow regime and complicated interactions between phases. In a nuclear power plant, two-phase flow is encountered in accident conditions, and the cooling performance of safety systems to mitigate the consequences is governed by the flow characteristics of a water-steam mixture. The key parameters used to describe the basic two-phase behavior include the void fraction and the flow velocity. In particular, the void fraction is a crucial variable used not only in predicting the transition of flow regimes but also heat transfer and pressure drop of two-phase flows, especially by means of an empirical approach. For this reason, versatile measurement techniques have been developed and applied to two-phase flow: including electrical [1,2,3,4,5,6,7,8,9,10,11], optical [12,13], ultrasonic [14] and radiation attenuation methods [15,16] and so on.

Among the categories outlined above, the electrical impedance technique has benefits in terms of a non-intrusive insertion into flow fields, easy application to various channel shapes, fast data acquisition rates, and convenient mobility. Classified as conductance methods and capacitance ones, the impedance technique using non-intrusive devices estimates the void fraction from measured electrical signals by employing a calibration curve that describes the relationship between the electrical impedance and the volume ratio of a phase. A variety of configurations for the electrode pairs have been proposed, including the plate-, ring-, helical-, and wire-type sensors. Of the two options, conductance sensors are applicable for a conducting medium when the frequency of the AC (alternating current) excitation signal is sufficiently high, i.e., 10~100 kHz for tap water [3].

We note that the electrical signals of an electrical impedance sensor depend not only on the area-averaged factions of a phase but also on the phase distribution inside the flow channel. The impedivity distribution within the domain can be reconstructed via Electrical Impedance Tomography (EIT) using electric fields [4,5,6], but this requires a complicated inversion algorithm process to process a large number of data, which is fairly time-consuming. Instead, by preparing a priori the calibration curve for representative flow structures in the region of interest, the conductance sensor is easily capable of measuring spatially averaged flow properties with high frequency responses. Extensive experimental and numerical studies have been conducted in order to investigate the electrical responses of a conductance sensor with a specific geometry, to determine the optimum configurations of electrodes, and to assess the capability of the proposed sensor in real multi-phase flow conditions [3,7,8,9].

Recently, inspired by the prior work by De Kerpel et al. [10], Ko et al. suggested an electrical conductance sensor that employs three concave electrodes for void fraction measurement in a horizontal pipe [11]. Using the measured conductance in the electrode pairs, the proposed sensor determined the flow regime of a two-phase flow through predetermined criteria for flow pattern classification and successively estimated the area-averaged void fraction from a relevant calibration curve. The measurement accuracy for the void fraction was validated against obtained data with the wire-mesh sensor. However, the proposed sensor was able to measure only the void fraction, and its application was limited to a basic horizontal pipe.

In this study, an improved electrical conductance sensor with a dual layer three-electrode set was proposed for the first time for simultaneous measurement of the area-averaged void fraction and the structure velocity, which is defined as the propagation velocity of the liquid-gas interface, of a two-phase flow. In particular, the proposed sensor was aimed at being used in inclined pipes, as well as horizontal ones. The industrial importance of the inclined pipe lies in the fact that, for a water-steam mixture under condensing conditions, it provides effective means to inhibit the formation of a water hammer by enabling effective drainage of the condensate water [17]. That is, the liquid flow is more accelerated in an inclined channel by the combined effect of an inclination angle and the gravitational force, which results in some differences in the flow structures from those in a horizontal channel. Thus, the applicability of the proposed conductance sensor to an inclined pipe needs to be evaluated.

On the basis of the numerical simulation, the electrodes configuration of each layer was determined the same as the design by Ko et al. [11], and the conductance sensor with a dual layer was fabricated. With reference to stratified, intermittent, and annular flow regimes in a downward inclined pipe, the conductance sensor was calibrated through a series of static experiments. The conductance sensor was applied to the U-shaped pipe with an inclination of 3° in which an adiabatic two-phase flow field is established, and the estimated void fraction and the structure velocity were compared to measured results using the wire-mesh sensor and using the visualized images recorded by the high-speed camera, respectively.

## 2. Sensor Design and Verification

### 2.1. Mathematical Background

**Figure 1:**
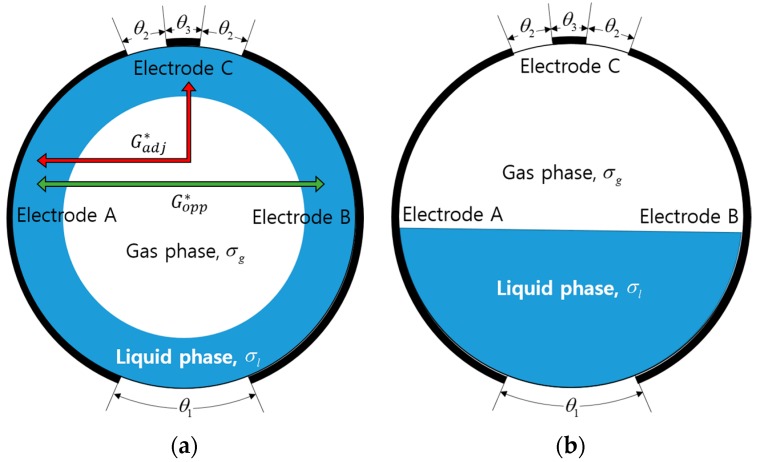
Configuration of electrodes in the proposed conductance sensor and typical flow structures considered: (**a**) Annular flow condition; (**b**) Stratified flow condition.

**Figure 2:**
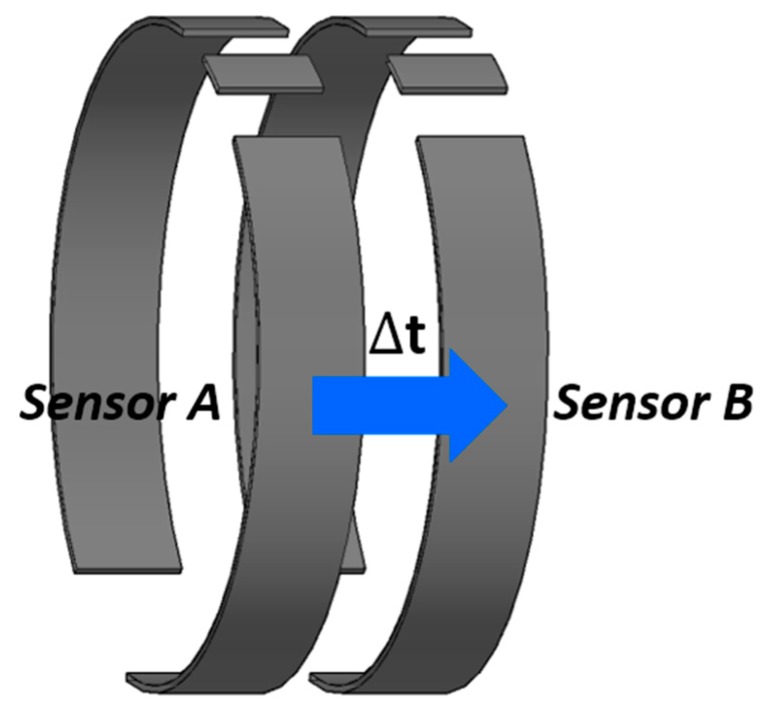
An improved dual conductance sensor system for measurement of the structure velocity.

The fundamental concept of the proposed conductance sensor is illustrated in Figure 1. Each layer consists of three concave electrodes arranged along the circumference of the circular pipe. The measured conductance between the adjacent electrode pair (electrodes A and C) is used to identify the flow regime of a two-phase flow. It takes into account the difference in the flow structures such that the whole circumference of the sensor will be in contact with a conductive liquid film in the annular flow, while the continuous liquid will wet partially the opposite electrode pair (electrodes A and B) without a contact to the electrode C in the stratified flow. The intermittent flow will exhibit periodic and repetitive readings of the electrical conductance by the liquid slug. Once the time-dependent electrical signals from two successive layers of electrodes are obtained, as depicted in Figure 2, the time lag between the layers is found through a correlation analysis to estimate the structure velocity of a two-phase mixture, which will be discussed in detail in Section 3.1.

In an air-water two-phase flow condition, the distribution of the electrical potential within the domain is described by the generalized Laplace equation as:

(1) ∇⋅(σ+iωε)∇u=0

where σ and ε denote the electrical conductivity and permittivity, respectively, and u represents the electrical potential to be determined for each phase. In cases when the conductivity component of a medium is much larger than the permittivity, then the reactance has little influence on the impedance, and Equation (1) reduces to:

(2) ∇⋅σ∇u=0

Then, the generalized Laplace equation for each phase of the air-water mixture is written as:

(3a) $$\nabla \cdot \sigma_{g}\nabla u_{g}=0\;\mathrm{for}\;\mathrm{gas}\;\mathrm{phase}$$

(3b) $$\nabla \cdot \sigma_{l}\nabla u_{l}=0\;\mathrm{for}\;\mathrm{liquid}\;\mathrm{phase}$$

If the current *I* flows between the electrodes of the sensor when the applied voltage difference is equal to ΔV, the electrical conductance is expressed as *G* = *I*/ΔV. In order to normalize the electrical conductance in a variety of sizes of sensors, a dimensionless conductance can be introduced as follows:

(4) $$G^{*}=\frac{G}{G_{l}}$$

where *G_l_* denotes the measured conductance value between the electrodes when the measurement domain is completely filled with the liquid at an ambient temperature, and thus the void fraction is zero.

### 2.2. Numerical Calculations for Sensor Design

The circumferential size of electrodes and the gap between them were determined by solving numerically Equation (3) in a circular domain. In this study, 3-D numerical calculation based on the finite element method was conducted using COMSOL Multiphysics. The angular gap between electrodes A and B in the bottom was denoted as $\theta_{1}$, and the circumferential size of the electrode C and the angle between electrodes at the top were represented as $\theta_{2}$ and $\theta_{3}$, respectively. They were determined by the requirement that the non-linearity error for the void fraction measurement be minimized in order to obtain a linear response to the variations of the gas or liquid volume fraction. The non-linearity error was defined as:

(5) $$\eta ={\left|G_{linear}^{*}-G_{opp}^{*}\right|}_{\max}\times 100\;(\%)$$

where $G_{linear}^{*}$ indicates the linear conductance, which has the same value as the liquid volume fraction, and $G_{opp}^{*}$ means the calculated dimensionless conductance between the opposite electrodes A and B for a given void fraction and geometrical parameters.

The numerical calculations reproduced the typical interfacial structure for the annular and the stratified flow regimes, and a wide range of the void fraction for each flow pattern was covered. The electrical properties of the medium and the dimension of the sensor used in the computation were summarized in Table 1. From the calculation results, the optimized design for the three-electrode sensor was decided as $\theta_{1}$ = 0.5 rad, $\theta_{2}$ = 0.2 rad, and $\theta_{3}$ = 0.3 rad. The non-linearity error was 5.7% for the annular flow, and 12.7% for the stratified flow. See Ko et al. [11] for details of simulation results.

**Figure 3:**
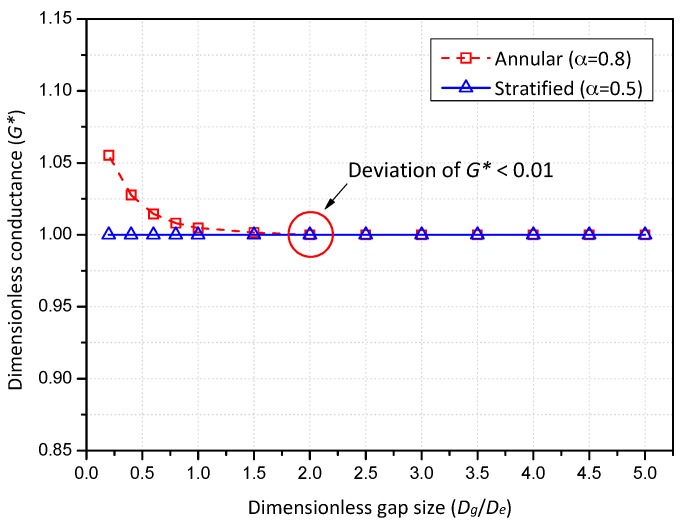
Variation of the calculated conductance according to the spacing between layers.

Another crucial parameter to be determined is the spacing between the electrode layers. If they are positioned too close, the electrodes in one layer may interfere with the measured electrical signals of another layer. On the contrary, when they are far apart from each other, the flow structure may vary substantially between them and the accuracy of the velocity measurement might be reduced. In order to determine a minimum distance that excludes the electrical interferences between layers, the variation of the conductance with the spacing between the layers was numerically simulated. The deviation from the dimensionless conductance for a single-layered sensor according to the spacing was plotted in Figure 3. Note that the *X*-axis of Figure 3 indicates the dimensionless spacing (*D_g_*/*D_e_*) defined as the spacing (*D_g_*) divided by the width of the electrode (*D_e_*), 15 mm. The simulations were carried out with regard to two flow conditions: two-phase conditions with the void fraction of 0.8 and 0.5 in the annular and stratified flows, respectively.

Inspection of Figure 3 revealed that the conductance sensor suffered from electrical interferences under single-phase liquid or stratified flow conditions as the spacing became narrow while the electrical conductance was rarely influenced by the annular flow spacing. As the dimensionless spacing of the dual sensor increases, the deviation from the conductance calculated for a single-layered sensor diminishes. The deviation of the dimensionless conductance was reduced below 1% when the dimensionless spacing was not less than 2.0. From the numerical results, the distance between the electrodes layers was determined to be 30 mm. For other diameters or inclination angles of a pipe, the design of the conductance sensor can be determined in the same way as the above mentioned optimization process. Depending on the flow structures in a targeted near-horizontal pipe, the configuration may be altered from the present one a little.

### 2.3. Verification

The electrical conductance sensor was fabricated based on the optimized design through the numerical calculations. The details of the sensor configurations are summarized in Table 2. The electrodes were flush-mounted on the inner wall of a short acrylic pipe unit.

**Figure 4:**
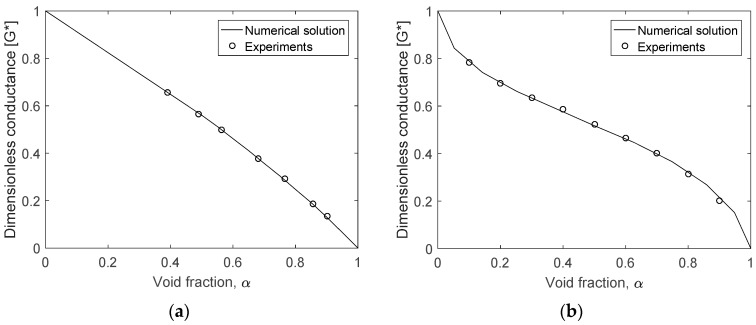
Predicted and measured dimensionless conductance as a function of the void fraction: (**a**) Annular flow; (**b**) Stratified flow.

The measurement accuracy of the fabricated dual conductance sensor was verified through a series of static experiments. The fractions of a phase inside a horizontal pipe were adjusted with stagnant water and acrylic rods and the linearity degree of the measured electrical conductance was compared to the predictions of numerical calculations. In simulating the annular flow, an acrylic rod whose diameter corresponds to a prescribed void fraction was placed at the center of the channel filled with water. The void fraction in the stratified flow was set by introducing known water volumes inside the horizontal pipe. The potential difference of 5 V was imposed to a pair of opposite electrodes A and B, and the frequency of the applied signal was set to 10 kHz. Refer to Section 3.2 for detailed information of the measurement system. The static experiments were performed for the void fraction in the ranges between 0.4 and 1.0 for the annular flow, and 0.0 and 1.0 for the stratified flow, respectively. Figure 4 presents the measured dimensionless conductance according to the void fraction, and the comparison results with the numerically obtained curve.

The profile of the dimensionless conductance exhibited a non-linear pattern with the variation of the void fraction. For both flow regimes, the measurements and the numerical value were in very close agreement over the void fraction range investigated. The measured non-linearity error was 7.0% for the annular flow, and 12% for the stratified flow, which were similar to those from numerical calculations. This demonstrated the adopted approach was reliable, and the conductance sensor was fabricated well following the intended design. The measured conductance data were then used in a form of the look-up table to determine the void fraction in subsequent flow conditions.

## 3. Experiments

### 3.1. Measurement Techniques

#### 3.1.1. Measurement of Void Fraction

In order to evaluate the void fraction of a two-phase flow using the conductance sensor, the flow pattern in the channel has to be identified at first. The flow patterns in a slightly inclined pipe are divided roughly into three conditions: stratified, intermittent, and annular flows. By taking into account typical flow patterns inside the flow channel and characteristics of corresponding electrical signals from three electrodes, one can distinguish the flow pattern established through the conductance sensor.

In a stratified flow, the phases are separated by the density difference. Thus water occupies the lower section of the cross-sectional height of the pipe while steam or air flows floating on water. As the highly conductive liquid rarely makes contact to the electrode C on the top of the flow channel, the dimensionless conductance measured between the adjacent electrodes A and C ($G_{adj}^{*}$) are very small. The static experiment revealed that the measured value in a simulated stratified flow condition was less than 0.005, which corresponded to the measurement error of the LCR meter used in the test. In actual flow conditions, however, dispersed water droplets or an incomplete liquid film around the periphery may affect the measured conductance. Thus, the upper threshold of $G_{adj}^{*}$ to identify the stratified flow was determined to 0.01.

As the transition into the intermittent or annular flow regimes occurs, the conductive water wets the electrode C on the top in a form of either thin liquid film or slug. Then $G_{adj}^{*}$ over 0.01 will be measured between the adjacent electrodes. According to the past studies, the annular flow appears when the void fraction is higher than 0.8 in an inclined pipe [18,19], which indicates that the dimensionless conductance between the opposite electrodes A and B ($G_{opp}^{*}$) will be lower than 0.30. Therefore, the upper threshold of $G_{opp}^{*}$ for the annular flow was set to 0.30. All subtle flow patterns between the stratified and the annular flows can be classified into an intermittent zone. Since the flow structure of the intermittent flow changes rapidly, but somewhat periodically, the conductance signal between the electrodes is expected to undergo the time-dependent fluctuations. Table 3 summarized the discussed criteria for flow pattern classification used in this work for an inclined pipe. Since the developed sensor uses dimensionless conductance values in classifying the flow structure and estimating the void fraction, this conductance sensor is applicable to any circular pipe unless the flow regime deviates significantly from the present cases.

Once the flow pattern is identified based on the criteria in Table 3, the void fraction is evaluated from the measured conductance between the opposite electrodes A and B. That is, the void fraction corresponding to the measured value of $G_{opp}^{*}$ is found using the calibration curve presented in Figure 4. For the intermittent flow, the complicated structure of the interface between phases makes it difficult to obtain the unique calibration curve from both numerical and experimental works.

This study assumed that the void fraction of the intermittent flow can be evaluated by employing the calibration curve of the stratified flow. With regard to this simplified approach, one needs to refer to the study of Roitberg et al. [20] which investigated the characteristic parameters of downward slug flow by means of the wire-mesh sensor. They presented that the annular-like domain of the elongated bubble's nose and tail, where some error is caused by the calibration curve of the stratified flow, is much shorter in length than the stratified-like domain of bubble’s body and liquid slug. Thus, even though not perfect, applying the calibration curve of the stratified flow to the intermittent flow regime does not cause significant error in the void fraction measurement.

#### 3.1.2. Measurement of Structure Velocity

The structure velocity of a two-phase flow is evaluated by the cross-correlation analysis of two area-averaged void fraction time series obtained from the dual conductance sensor. From the time-varying void fraction records, one finds the time-shift that maximizes the Pearson correlation coefficient; it describes the degree to which two parameters are linearly correlated. The Pearson correlation coefficient gives values between 1 and −1, where a value of 1 implies a perfect linear relationship between parameters, and 0 indicates no linear correlation. As the time series of void fraction signals are obtained from two successive layers of the electrodes, the time lag elapsed for a two-phase flow to move between the layers is determined by the requirement that the correlation coefficient be maximum. Then the structure velocity of a two-phase flow is calculated as follows:

(6) $$j_{sv}=\frac{D_{g}}{\Delta t}$$

where *D_g_* is the spacing between the layers (30 mm in this study), and Δt is the time lag that gives the maximum cross-correlation coefficient. Note that the velocity by Equation (6) is not a phasic velocity, but the propagation velocity of the interfacial structures between phases [21]. For example, the structure velocity corresponds to the propagation velocity of the periodic surface of liquid disturbance waves for the stratified-wavy flow, and that of highly unstable films for the annular flow. For the slug or elongated bubbles flow, it corresponds to the velocity of the large gas bubbles since the phasic fraction in the flow channel is determined by them. Note that this structure velocity is governed by the bulk structure of the phases, and the dispersed phase affects little to it. By the principle, the velocity measurements with this conductance sensor are limited to the axial direction of the pipe.

### 3.2. Experimental Setup

**Figure 5:**
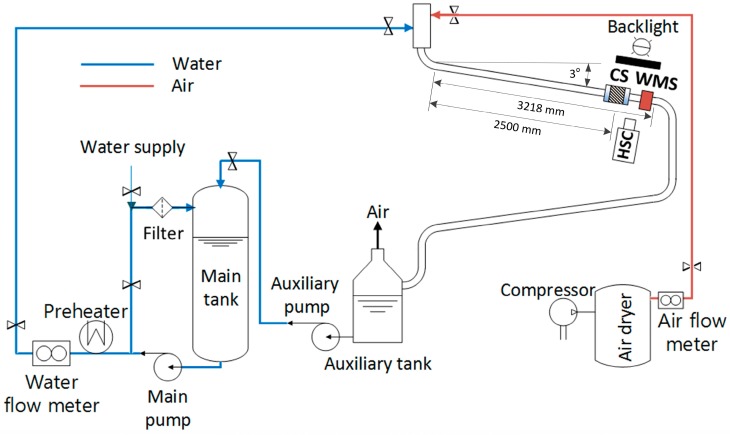
Schematic diagram of the air-water two-phase flow loop.

Illustrated in Figure 5 is the schematic diagram of the air-water two-phase flow loop at Jeju National University. This experimental facility consists of the main tank, the main pump, the preheater, the air compressor, the test section, and the separator. The flow rate of the supplied water to the test section is controlled by the main pump, and measured by the Coriolis flowmeter installed downstream of the main pump. The compressed air is supplied to the injector located near the entrance of the test section and mixed with water. Its flow rate is also measured by the Coriolis flowmeter. After flowing through the test section, a two-phase mixture is separated at the collection tank where air is discharged to the atmosphere and water is recirculated to the main tank.

The test section is the U-shaped inclined pipe with an inclination angle of 3°. The test section was constructed to simulate the heat exchanger of the Passive Auxiliary Feedwater System (PAFS) adopted in a next-generation Korean nuclear power plant, APR+ (Advanced Power Reactor Plus). It removes passively the decay heat from the reactor core by condensing steam from the steam generator inside a heat exchanger submerged in a large pool [22].

**Figure 6:**
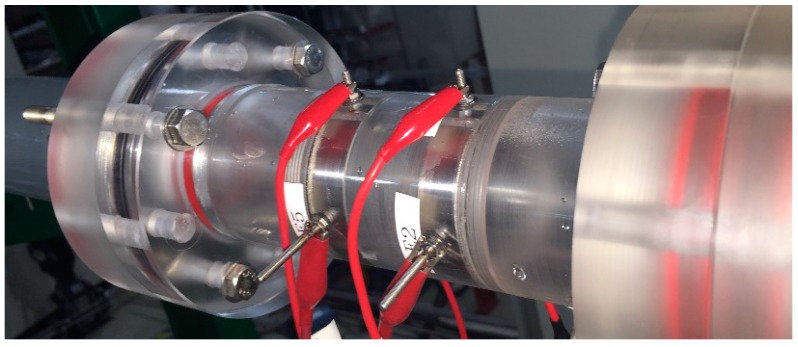
Schematic diagram of the air-water two-phase flow loop.

To prevent the occurrence of a condensation-induced water hammer inside the tube, the heat exchanger was designed to be inclined at 3°. The test section of the experimental facility is made from acrylic pipes to enable visual observation of a two-phase flow inside the flow channel. It is 44.8 mm in inner diameter, and 7246 mm in total length including the bend. The length of the straight test section is 3218 mm. The air-water two-phase mixture flows downward along this slightly inclined pipe. The proposed conductance sensor was placed at a distance 2500 mm from the entrance of the test section (*L*/*D* = 55). A photograph of the dual conductance sensor installed in the test section is shown in Figure 6.

**Figure 7:**
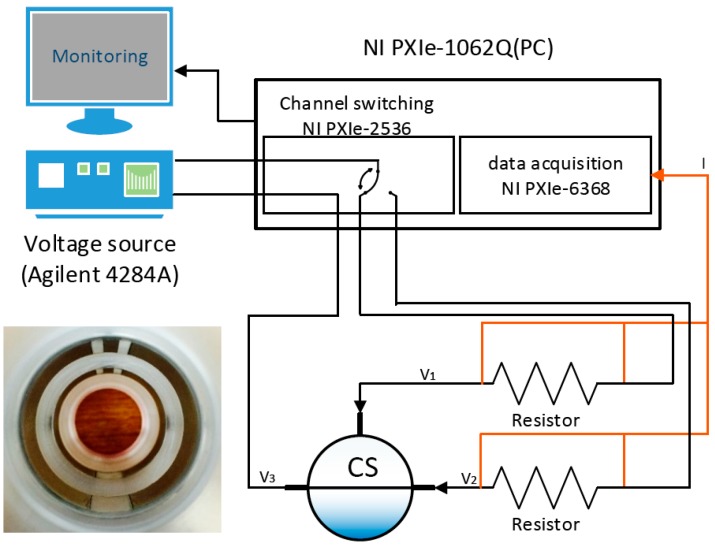
Measurement system for the conductance sensor.

Figure 7 presents the schematic diagram of the measurement system for the conductance sensor. An AC voltage was applied via a 4284A LCR meter (Agilent technologies, Santa Clara, CA, USA) to a pair of electrodes. The switch module (NI PXI-2536, National Instruments, Austin, TX, USA) then opened and closed alternatively the circuits connected to the opposite and the adjacent pairs of electrodes, respectively. A shunt resistor was connected in parallel between the electrode and the switch module. The voltage difference across the resistor was measured by a DAQ unit (NI PXI-6368, National Instruments, Austin, TX, USA) and converted to the current signal. Detailed specifications of the devices used in the measurement are shown in Table 4. The collected conductance signal passing through the circuit was processed in real time to calculate the void fraction according to the results of the flow regime classification.

The applied voltage was set to 5 V with a signal frequency of 10 kHz to ensure that the impedance response became conductive. As the evaluation of the flow pattern and the void fraction was carried out alternatively during the switching frequency, the maximum measurement speed of the proposed conductance sensor was 5000 fps. In the experiment, it was adjusted to 500 fps in consideration of the accuracy of the data acquisition. The switching speed and the sampling rate was set to 2 kHz and 2000 kps, respectively.

### 3.3. Test Matrix

**Figure 8:**
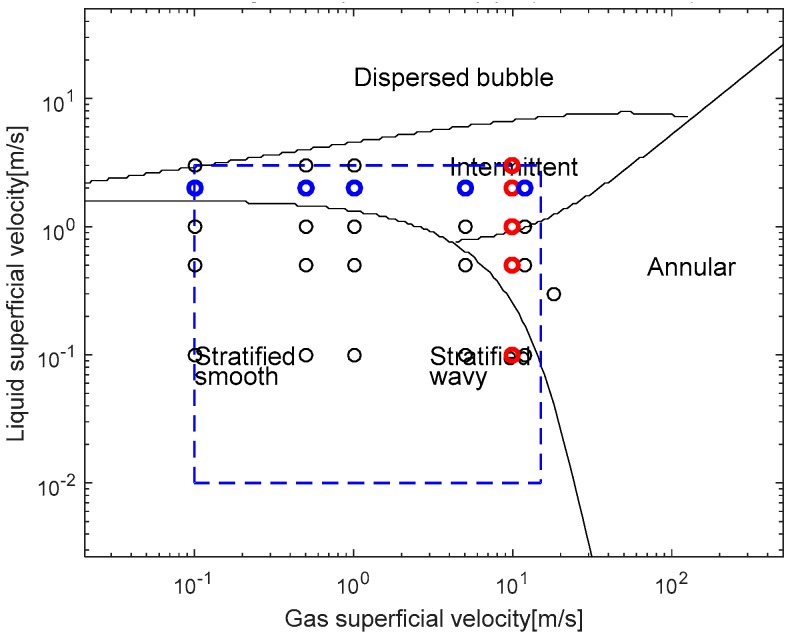
Investigated flow conditions marked on the flow regime map by Taitel and Dukler [23] (the colored marks indicate the selected runs to be discussed in Section 4.2. Measurement Results. The reds are to investigate the effect of the liquid velocity at a fixed gas velocity, whereas the blues indicates tests performed at a fixed liquid velocity with varying the gas velocity. The dash-line box encloses the testable ranges in the JNU two-phase flow loop).

The developed conductance sensor system was applied to a series of the air-water two-phase flow experiments. A wide variety of superficial velocities ranging from 0.1 to 3.0 m/s for water and from 0.1 to 18 m/s for air were tested. Some selected flow conditions discussed herein are summarized in Table 5, and marked on the flow regime map by Taitel and Dukler [23] for near horizontal two-phase flows in Figure 8. The fluid temperature was kept constant at an ambient temperature of 22 °C to 24 °C during the experiments. Due to the limited capacity of the air compressor used in this experiment, a complete form of an annular flow could not be established; however, one expected the sporadic appearance of an annular liquid film around the periphery of the flow channel in the intermittent flow conditions.

## 4. Results and Discussion

### 4.1. Validation

**Figure 9:**
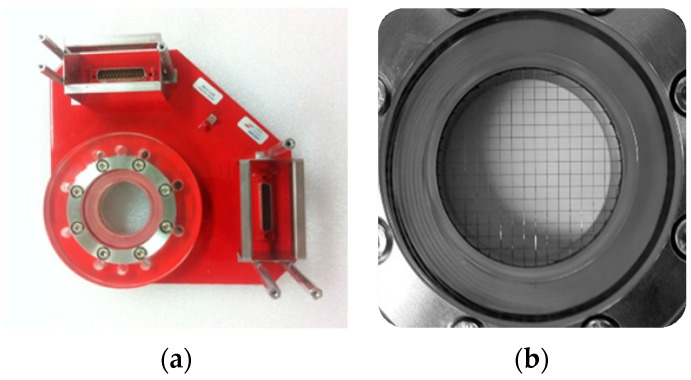
Wire-mesh sensor manufactured by HZDR Inc. (Dresden, Germany) for validation of the measured void fraction. (**a**) Photograph of the wire-mesh sensor used in this study. (**b**) Transmitter and receiver electrodes mesh of the wire-mesh sensor.

The measured void fraction and structure velocity by the conductance sensor was validated using other independent measurement techniques. The obtained test results for the void fraction were assessed against the measured value by the wire-mesh sensor. The wire-mesh sensor, a commercial sensor developed by HZDR Inc. (Dresden, Germany), measures the void fraction using the difference in the electrical conductivity of the employed fluids [24]. It consists of two planes of wire grids placed into the flow: the transmitter and receiver planes as shown in Figure 9. The wires of both grids are perpendicular, thereby creating the rectangular meshes. Thus the wire-mesh sensor enables measuring the instantaneous local volumetric ratio of a phase at all crossing points of wires of the two planes. The measurement frequency of the wire-mesh sensor was 10,000 fps in the tests.

The wire-mesh sensor used in this study had 16 × 16 grid configuration, thus with 256 intersections of wires. The thickness of the steel wire was 0.1 mm, and the two planes of wires were apart from each other by 2.5 mm. The wire-mesh sensor was placed at a distance 200 mm downstream from the conductance sensor. This spacing was the best compromise obtained from the numerical simulation to exclude the influence of electrical interferences between the sensors and to minimize the variation in the phase distribution while passing through both sensors.

**Figure 10:**
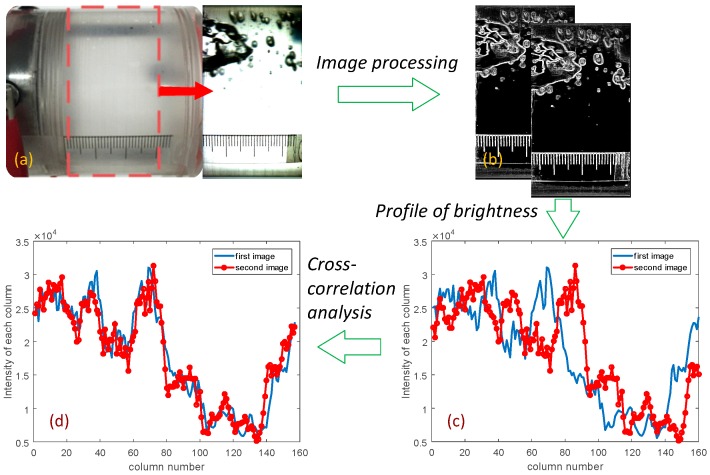
Cross-correlation method using the visualized images for verification of the measured structure velocity. (**a**) Visualized image recorded by the high-speed camera. (**b**) Phase edge detection applied to two images captured with a known time interval. (**c**) Distributions of the brightness integrated by column. (**d**) Shift in distance determined by the cross-correlation analysis for the best match of the brightness distributions.

The structure velocity measured by the conductance sensor was validated using the visualized images recorded by the high-speed camera. Figure 10 illustrates the process to estimate the structure velocity by the high-speed camera measurement technique. The flow field inside the inclined pipe was visualized from the side using back-lighting, which created the shadow along the interfaces of air bubbles. Then the boundaries between phases were discriminated precisely by the Sobel edge detection scheme provided by the image analysis tool, MATLAB (MathWorks, Natick, MA, USA) (Figure 10b). The local brightness of the processed image was determined by the phase distribution. Then, one divided finely the entire image into the flow direction to set the regions of interest and calculated the sum of values of brightness in each area so that their profile across the image could be plotted as the blue line in Figure 10c. The same process was repeated for the image captured after a few frames, and then almost the same transversal profile of the sum of brightness can be extracted with a slight shift in distance. By applying the cross-correlation technique to two consecutive images information, one can trace the moving distance of the interfaces during a constant time interval.

The procedure mentioned above was applied to a couple of pairs of sequential images to estimate the average structure velocity of a two-phase flow. In short, this high-speed camera measurement technique estimates the structure velocity from the distance that the interface moved during a time interval, whereas the conductance sensor calculates the time lag elapsed to move a fixed distance.

**Figure 11:**
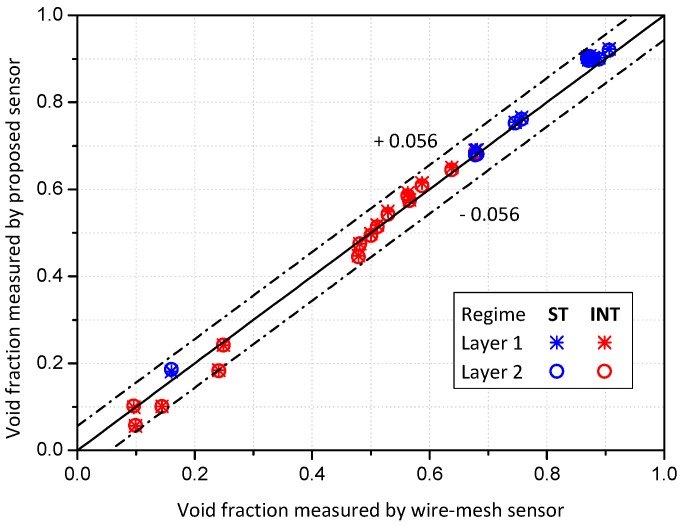
Comparison between the time-averaged void fractions measured by the proposed sensor and the wire-mesh sensor (note: ST and INT stand for the stratified and intermittent flow regimes, respectively).

The comparison results of the time-averaged void fraction measured from the conductance sensor and the wire-mesh sensor are presented in Figure 11. The measured void factions from the proposed conductance sensor were in very good agreement with those from the wire-mesh sensor. The deviation of the void fractions by two sensors were less than 5.6%, and the average absolute error was about 2.0%.

Inspection of Figure 11 reveals that, in high liquid fraction regimes, the conductance sensor tended to under-estimate the void fraction a little compared to the measured value via the wire-mesh sensor. At high liquid flow rates, discrete small bubbles are suspended in a continuous liquid phase. Since the conductance sensor was based on the electrical signal passing through the whole cross-sectional area of the pipe, the effect of dispersed bubbles or liquid drops with a small volume fraction may not be captured precisely. In addition, the effect of liquid film surrounding the elongated bubble in the intermittent flow may be not considered possibly due to the application of the calibration curve of the stratified flow to the intermittent flow regime. Nevertheless, the deviation stayed within an acceptable range, and thus it was proved that, even though non-intrusive, the developed conductance sensor can provide reliable measurement for the void fraction of a two-phase flow in an inclined pipe.

Table 5 presents the measured structure velocity via the conductance sensor at five flow conditions. Note that the experiments to validate the measured structure velocity were conducted separately to establish the flow conditions to which the high-speed camera measurement technique described by Figure 10 was readily applicable. All five runs in Table 6 were conducted at the intermittent flow regime.

Thus the measured structure velocity corresponds to the air-phase interfacial velocity of the slug or elongated bubble flow. The comparison results in Table 6 revealed that the deviation between the structure velocity measured by the conductance sensor and that estimated using the high-speed camera was less than 1.6%. It indicated that the proposed sensor could also measure the structure velocity with high accuracy.

### 4.2. Measurement Results

**Figure 12:**
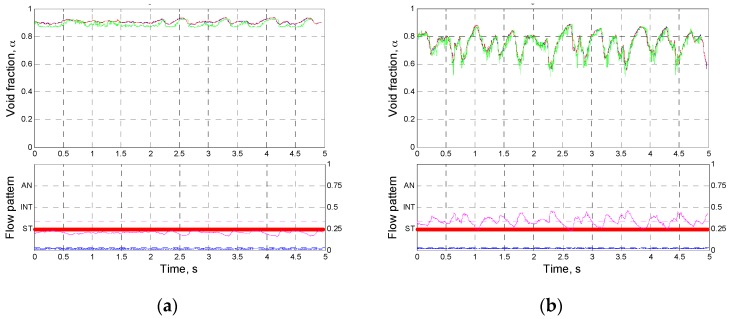

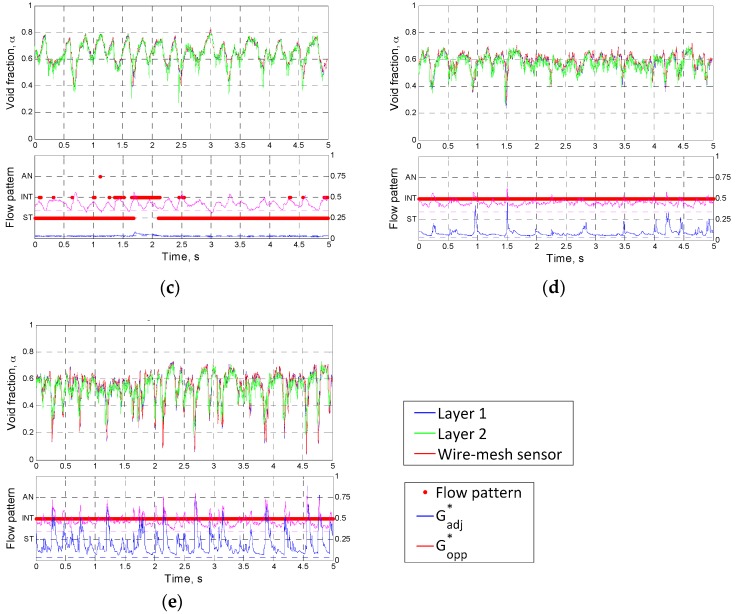
The results of the flow pattern classification and the corresponding time series of the void fraction with varying the liquid superficial velocity (Note: The right *Y*-axis of the lower graphs indicates the dimensionless conductance.) (**a**) Case 1: *j_l_* = 0.1 m/s, *j_g_* = 10 m/s, *j_sv_* = 1.18 m/s. (**b**) Case 2: *j_l_* = 0.5 m/s, *j_g_* = 10 m/s, *j_sv_* = 1.96 m/s. (**c**) Case 3: *j_l_* = 1.0 m/s, *j_g_* = 10 m/s, *j_sv_* = 2.77 m/s. (**d**) Case 4: *j_l_* = 2.0 m/s, *j_g_* = 10 m/s, *j_sv_* = 4.27 m/s. (**e**) Case 5: *j_l_* = 3.0 m/s, *j_g_* = 10 m/s, *j_sv_* = 6.38 m/s.

**Figure 13:**
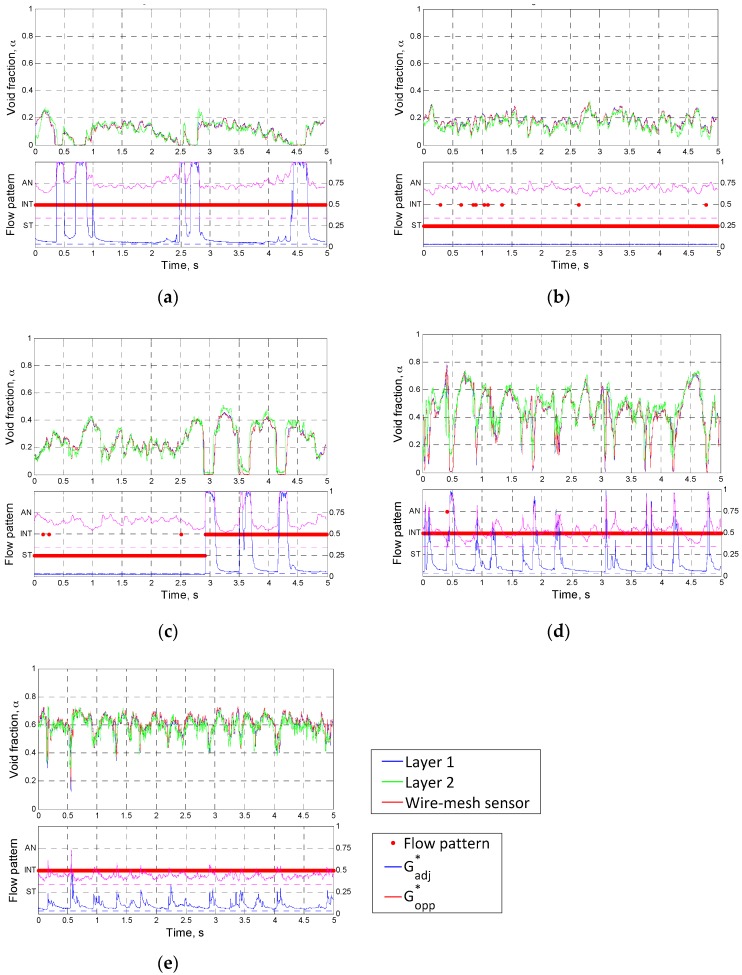
The results of the flow pattern classification and the corresponding time series of the void fraction with varying the gas superficial velocity (Note: The right *Y*-axis of the lower graphs indicates the dimensionless conductance). (**a**) Case 6: *j_l_* = 2.0 m/s, *j_g_* = 0.1 m/s, *j_sv_* = 2.04 m/s. (**b**) Case 7: *j_l_* = 2.0 m/s, *j_g_* = 0.5 m/s, *j_sv_* = 2.29 m/s. (**c**) Case 8: *j_l_* = 2.0 m/s, *j_g_* = 1.0 m/s, *j_sv_* = 2.49 m/s. (**d**) Case 9: *j_l_* = 2.0 m/s, *j_g_* = 5.0 m/s, *j_sv_* = 4.05 m/s. (**e**) Case 10: *j_l_* = 2.0 m/s, *j_g_* = 12 m/s, *j_sv_* = 4.91 m/s.

Based on the validation works for the proposed conductance sensor, the cross-sectionally averaged time series of the void fraction in the inclined pipe were plotted in Figure 12 and Figure 13. The results of the flow pattern classification by the criteria in Table 3 and the measured void fraction from the wire-mesh sensor were also included in these figures. Note that the measured signal from the electrode layer at the rear was time-shifted by using the estimated structure velocity. Inspection of Figure 12 and Figure 13 revealed that the time-varying void fractions from two layers of electrodes agreed very well. This implies that the variation in the flow behavior of the air-water two-phase flow was negligible in the interval between the layers, and the estimated structure velocities from the conductance sensor were accurate.

Figure 12 presents the measurement results of the time-varying void fraction with a change in the liquid superficial velocity at a fixed gas superficial velocity of 10 m/s. At a very low liquid superficial velocity (case 1), a stratified-wavy flow was sustained stably at a high-void fraction region. At higher gas superficial velocity, the wavy interfaces of the liquid grew large (case 2) and the liquid slugs appeared to cause large fluctuations in the time series of the void fraction (case 3). Further increase of the liquid velocity lowered the mean void fraction and transited the flow regime to complete intermittent flow. In cases 4 and 5, some finite value of $G_{adj}^{*}$ was measured continuously, indicating that a thick liquid film developed near the top of the flow channel.

Referring to the test results of Ko et al. [11] obtained in a horizontal pipe, one may notice that the length of the liquid slugs was relatively short, which was recognized from high fluctuation frequency of the void fraction signal. This attributed to the acceleration of the liquid phase in the inclined channel under the influence of the gravitational force. It is also worthy of noting that, even though the gas superficial velocity in this experiment was generally higher than that in the tests of Ko et al., the two-phase flow pattern classified to the annular flow hardly appeared, even instantaneously.

At a constant liquid superficial velocity of 2.0 m/s, the measured time series of the void fraction with varying the gas superficial velocity were plotted in Figure 13. The increase in the gas flow rate lead to gradual rise of the mean void fraction. When the gas superficial velocity was very low in case 6, almost entire cross section was occupied by the liquid phase, and corresponding high value of $G_{adj}^{*}$ made the flow regime be identified into the intermittent one. A slight increase in the gas flow rate rather formed stable stratified-wavy flow as case 7. When the gas superficial velocity increased up to 5.0 m/s, the two-phase flow was characterized by incessant passing of large waves and frequent oscillations of the interface. For the gas superficial velocity over 10 m/s, a thin liquid film was sustained on the pipe wall and thus the value of $G_{adj}^{*}$ larger than 0.01 was continuously detected. However, due to the low void fraction, the flow regime classified to the annular flow was not observed alike the experimental results in Figure 12.

The above measurement results demonstrated that, even though the developed conductance sensor was based on a non-intrusive approach, it could capture well the characteristics of the two-phase flow in an inclined channel through the collected electrical signals. Moreover, it provided practical information to distinguish the flow regime in the inclined pipe notwithstanding very simple criteria for the flow regime identification.

Figure 12 and Figure 13 also indicated that the measurement results of the time-varying void fractions using the developed conductance sensor were in very good agreement with those from the wire-mesh sensor. It was noted that, in the measurement results from the dynamic tests, the intermittent flow was observed in more broad ranges of the flow conditions than the domain defined in the flow pattern map by Taitel and Dukler of Figure 8.

**Figure 14:**
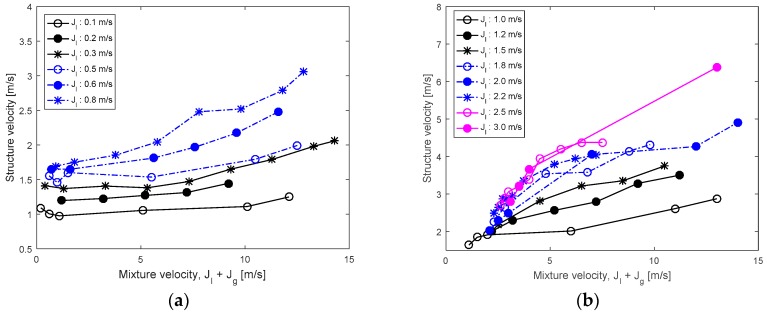
The relationship between the structure velocity and the mixture velocity: (**a**) *j_l_* = 0.1~0.8 m/s; (**b**) *j_l_* = 1.0~3.0 m/s.

The structure velocities are plotted against the mixture velocity, defined as the sum of the gas and liquid superficial velocities, in Figure 14. The plots show that an increase in the mixture velocity lead to a consistent increase of the structure velocity even if their relationship was not exactly linear. Once the liquid superficial velocity was very low, the velocity of disturbance waves changed little as the gas superficial velocity was augmented. In most flow conditions investigated, the structure velocity was lower than the mixture velocity; a few exceptions were noted when both of the gas and liquid superficial velocities were low since the liquid flow was accelerated by an inclination.

## 5. Conclusions

For application to the gas-liquid two-phase flows in an inclined pipe as well as a horizontal pipe, this study proposes for the first time an improved conductance sensor with a dual layer three-electrode set capable of measuring instantaneous cross-sectionally averaged void fractions and structure velocities. The conductance sensor was calibrated through a series of static experiments, and applied to the U-shaped downward pipe with an inclination of 3° under adiabatic air-water flow conditions. The measured mean void fraction and structure velocity were validated against the wire-mesh sensor and the cross-correlation technique for the visualized images, respectively. The estimated deviation was less than 5.6% for the void fraction, and 1.0% for the structure velocity.

The measurement results in the inclined loop revealed that the time series of the mean void fraction agreed very well with those from the wire-mesh sensor. Since the proposed conductance sensor requires no intrusive wires or probes into the flow fields, it did not disturb the structure of a two-phase mixture for an necessity for measurement. Nevertheless, it could capture well the characteristics of the two-phase flow in an inclined channel through the collected electrical signals from two pairs of electrodes. In addition, it can be fabricated readily and implemented easily to either a horizontal or an inclined orientation on account of simple configuration. Therefore, it is expected that the proposed conductance sensor can be a useful instruments for simultaneous measurement of the area-averaged void fraction and the structure velocity of a two-phase flow.

Unfortunately, even though the proposed conductance sensor was designed to be applicable for the annular flow regime as well, it could not be tested due to the limited capacity of the air supply in the experimental apparatus. Thus the criterion of the transition from intermittent to annular flow was also determined from the previous literature. Due to its measurement characteristics, the proposed sensor has some problems in detecting a small-fraction dispersed phase. To enhance the reliability of the conductance sensor, the uncertainty of the instantaneous void fraction measurement for the annular flow as well as the transition criteria from intermittent to annular flow need to be quantified through the dynamic tests. The improvement of the calibration procedure for the intermittent flow is also required as a further work.

## Figures and Tables

**Table 1:** Electrical properties and sensor dimensions for the numerical calculation.

Variables		Value
Conductivity (S/m)	Water	0.005
	Air	0
	Electrode	1.0 × 10^6^
	Gap	0
Applied voltage (V)		5
Signal frequency (kHz)		10
Inner diameter of the sensor (mm)		45
Width of electrodes (*D_e_*, mm)		15
Dimensionless gap size (*D_g_*/*D_e_*)		0.25~5

**Table 2:** Details of the optimized sensor configurations.

Variables		Value
Circumferential size (rad)	Electrode A & B	2.54
	Electrode C	0.30
Inner diameter of the sensor (mm)		45
Width of electrodes (*D_e_*, mm)		15
Thickness of electrodes (mm)		2 (concave)
Spacing between layers (mm)		30

**Table 3:** Criteria for flow pattern classification.

$G_{adj}^{*}$	$G_{opp}^{*}$	Flow Regime
<0.01	-	Stratified flow
≥0.01	<0.3 (α>0.8)	Annular flow
	≥0.3 (α≤0.8)	Intermittent flow

**Table 4:** Specifications of measurement instruments used for experiments.

Instruments	Accuracy	Signal Range	Time Definition
Agilent 4284A LCR meter	0.05~0.5%	Up to 20 V with 1 MHz	N/A
NI PXI-2536	N/A	Up to ±12 V and 100 mA	5 × 10^4^ cross-points/s
NI PXIe-6368	3 mV for ±10 V range	Up to ±10 V	2 × 10^6^ samples/channel

**Table 5:** Test matrix for air-water two-phase flow experiments in the inclined loop.

Run	*j_l_* (m/s)	*j_g_* (m/s)	Flow Pattern “by Map”
1~5	0.1	0.1, 0.5, 1, 5, 10, 12	Stratified flow
6	0.3	18	Annular low
7~11	0.5	0.1, 0.5, 1, 5, 10, 12	Stratified flow
12~16	1		Intermittent flow
17~21	2		Intermittent flow
22~25	3	0.1, 0.5, 1, 10	Intermittent flow

**Table 6:** Validation results of the structure velocities measured by the conductance sensor.

Case	*j_l_* (m/s)	*j_g_* (m/s)	*j_sv_* (m/s)		Deviation
			Conductance Sensor	High Speed Camera	
A	1.8	5.0	3.58	3.60	−0.56%
B	2.2	0.3	2.65	2.62	1.2%
C	2.5	2.0	3.94	3.88	1.6%
D	3.0	0.5	3.19	3.16	0.95%
E	3.0	1.0	3.66	3.66	0%
